# Study on the strategy of multimodal transportation of medical aid materials for public health emergencies: a case study basing on COVID-19

**DOI:** 10.1093/ijlct/ctab046

**Published:** 2021-06-10

**Authors:** Tao Ning, Xiaodong Duan, Lu An

**Affiliations:** 1 Institute of Computer Science and Engineering, Dalian Minzu University, Dalian 116000, China; 2 Big Data Application Technology, Key Laboratory of State Ethnic Affairs Commission, Dalian 116000, China; 3 Institute of Software, Dalian Jiaotong University, Dalian 116021, China

**Keywords:** multimodal transportation, public health emergency, COVID-19, QBFwCC, route optimization

## Abstract

The emergency response to sudden public health incident often encounters problems such as the long distance between the medical material supply node and the demand point or the destruction of key roads, which makes it difficult to transport materials directly to the disaster area by vehicles in time. Helicopters are increasingly being used to transport critical medical emergency resources, but it is not easy to distribute medical supplies through helicopter in large-scale public health incidents. In order to solve the above problems, a two-stage combined transportation method for medical supplies based on clustering is proposed in this paper. In the first stage, the quantum bacterial foraging (QBF) algorithm is used to select emergency transit points and divide the medical assistance points. In view of the imbalance of remaining capacity in QBF division, an improved division method (quantum bacterial foraging with capacity constraints (QBFwCC)) is proposed and a ‘helicopter–vehicle’ medical material combined transportation network structure is constructed in consideration of capacity constraints. In the second stage, a transportation route optimization model based on clustering is established to determine the specific transportation route from the emergency transit point to the medical assistance point. The performance of the method proposed in the paper was analyzed through experimental COVID-19 simulation and compared between QBF and QBFwCC. The results show that the method not only achieves the goal of optimization, but also effectively reduces the number of vehicles.

## 1. INTRODUCTION

The timely delivery and rational distribution of relief materials in public health emergencies is one of the key contents of emergency research, which will damage the health and life of the affected people directly. Public health emergencies are closely related to environmental pollution, ecological destruction and traffic accidents. Those events have such features as distribution differences, time urgency, frequent incidents and serious consequences. It is difficult to distribute medical supplies directly by helicopter when a public health emergency occurs. Usually, a certain number of emergency distribution points (EDPs) are selected to receive medical supplies from helicopters, and then to distribute the supplies to the corresponding medical aid points (MAPs) by vehicles. However, there is a series of difficulties for combined transportation, such as how to choose the location of emergency transit points, how to divide MAPs into different EDPs and how to arrange transportation routes from EDPs to MAPs covered by them. Public health emergency medical materials are the material basis for the implementation of public health emergency plans. At present, scholars at home and abroad have studied the allocation and transportation of emergency medical resources from many perspectives such as scheduling strategy, solution algorithm and rescue route optimization. Feng *et al*. [4] considered the uncertainty of demand and the scarcity of supplies. They constructed a maximum coverage model for medical facilities location and designed three heuristic algorithms for model solving. Acheampong *et al*. [1] based on loss estimation model, considered the time throes, fairness and possible social unrest in the allocation of emergency medical supplies. The ambulance allocation model and relaxation algorithm with the shortest overall rescue time is studied, under the limited ambulances number. Ning *et al*. [12] designed a random planning model by using event scenarios to capture specific information to select the storage location and inventory level of emergency medical supplies. Arora *et al*. [2] proposed an emergency rescue resource allocation method for regional assistance in public health emergencies. Xiang *et al*. [14] designed a large-scale disaster relief material allocation method according to different event scenarios. Yarmand [15] proposed and tested a heuristic vaccine distribution method for two-stage random linear planning problem. Ning *et al*. [11] studied the traveling salesman model and vehicle routing optimization model of emergency medical supplies after major public health events, which optimized to minimize the maximum and average arrival times. Yang *et al*. [5] applied improved genetic optimization to dynamically allocate uncertainty emergency response and evacuation traffic flow problems with time-dependent demand. A system optimal dynamic allocation optimization model based on the cell transmission model is constructed and the affine adjustable robust correspondence algorithm is used to solve the problem in this paper. Chen *et al*. [3] designed a robust network optimization model for blood scheduling in-disaster and post-disaster that can be used for site selection and configuration decisions at multiple blood storage facilities. Ning [8] explored the allocation of the last mile for resource allocation and vehicle routing decision-making in Haiti’s public health emergencies. Ning *et al*. [7] designed a logistics decision support model that integrates the multi-material network flow problem and the vehicle routing problem. Dynamic time-varying transportation problem is solved by Lagrangian relaxation algorithm. Peng *et al*. [9] abstracted the container multimodal transport emergency rescue system as an approximate immune system network. An integer linear programming model is proposed, which is used to select the route of container supply chain in an emergency rescue environment. Qiang *et al*. [10] discussed the rescue problem that the World Food Programme used airplanes to urgently deliver food to Angola and established a location-routing problem (LRP) model. Yokokawa *et al*. [6] established a dynamic allocation model of emergency relief supplies, which was to optimize pre-disaster planning and applied to the hurricane raging incident in North Carolina. Wang *et al*. [13] proposed a non-dominated sorting genetic algorithm. A non-linear integer development LRP model with post-disaster delivery time, total cost and segmentation cost was established. Shi *et al*. [16] designed a variable neighborhood genetic search algorithm. Two-stage stochastic programming LRP model with emergency network failure, repeated use of vehicles and standard rescue time was proposed.

Most of the above studies explore the transport of a single material by a single distribution method. There are only a few studies focused on the distribution of multi-species relief supplies through multi-transport. However, there are few systematic studies on the distribution of emergency medical relief materials in multiple varieties and methods. In response to the actual problems in the above research, a two-stage combined transportation method of medical materials based on clustering is proposed, taking the COVID-19 incident as an example. In the first stage, emergency transit point selection and MAPs division of quantum bacterial foraging algorithm are proposed according to the distribution of MAPs. Considering the capacity constraints in each division for the imbalance of residual capacity in Quantum Bacterial Foraging (QBF) division, an improved QBF with capacity constraints algorithm is designed to optimize the division of MAPs, so as to construct a ‘helicopter–vehicle’ medical supplies combined multimodal network structure. In the second stage, a cluster-based transportation route optimization model is established, which can determine the specific transportation route from the emergency transit point to the MAPs.

The structure of this paper is arranged as follows: In Section 2, the problem of emergency response to sudden public health incident, its objective function and constraint are described. In Section 3, the heuristic algorithm based on QBF and QBFwCC emergency transition point selection and division are proposed. In Section 4, experimental data are set and the results are statistically analyzed to verify the effectiveness of proposed method. In Section 5, the strategy of multimodal transportation of medical aid materials for public health emergencies is concluded and further research directions are analyzed.

## 2. MATHEMATICS MODEL

### 2.1. Description of problem

The decision-making problem to be solved in this paper can be described as follows. In the COVID-19, the emergency management department has established *n* MAPs to provide medical aid to patients. Medical supplies need to be transported from other places to each MAP in the epidemic area as soon as possible. The emergency management department uses an airport in the area as a large collection and distribution hub to receive medical supplies sent from outside. In order to ensure that the medical supplies received by large-scale center for distribution hub (LCDH) are delivered to each MAP, the emergency management department sends a helicopter to transport the epidemic prevention supplies. However, due to the limited number of helicopters and the scattered distribution of epidemic areas, it is difficult to transport medical supplies to each MAP directly by helicopter. Establishing *m* EDPs to receive the materials delivered by helicopters and then using vehicles to transport medical materials from EDPs to each MAP are considered in this article.

The problem includes two sub-problems: the location of EDPs and the vehicle paths from the EDPs to each MAP. The goal of problem decision-making is to deliver the required medical supplies to each MAP in the shortest possible time, i.e. to minimize the delivery time of medical supplies. In order to define the study scope of application, the following assumptions were made: (1) All medical supplies needed in the epidemic area are first transported from the outside to LCDH, and then transported from LCDH to the EDPs by helicopter and finally transported from the EDPs to each MAP by vehicle. (2) The amount of medical supplies allocated to each MAP is known. (3) Each helicopter is responsible for delivering medical rescue supplies to an EDP each time, and each helicopter has the same capacity. (4) Each MAP is visited by the same vehicle only once. Vehicles of the same type (same capacity) depart from their respective emergency transit points, and finally return to the transfer station. (5) The distance from LCDH to each EDP and the distance from the EDP to the MAP are known.

### 2.2. Location selection of EDPs based on clustering

How to choose the location of the EDPs scientifically and reasonably is the first sub-problem to be solved. According to the location distribution of MAPs, a clustering method based on division to select the location of EDPs is used in this paper. QBF method is used for partition clustering. Firstly, EDPs are selected by using QBF method, and then an improved Quantum Bacterial Foraging with Capacity Constraints (QBFwCC) method considering capacity constraints is proposed for unbalanced capacity problem.

The corresponding relations of symbols used in the mathematical model of this article are as follows:

*n*: the number of MAPs in epidemic area;
*A_i_*: the *i* MAPs, *i* = 1,2,…，*n*;
*s*: the quantity of available medical aid materials in LCDH;
*d_i_*: the amount of medical supplies allocated by Ai at the MAP, *i* = 1,2,…,*n*;
*m*: the number of EDPs;
*C_j_*: the *j* EDPs, *j* = 1,2,…, *m*;

${N}^{C_j}$ : a set of MAPs covered by the EDP *C_j_*;

${n}^{C_j}$ : the number of elements in set ${N}^{C_j}$;

${N}_0^{C_j}$ : the set of ${N}^{C_j}$ EDP *C_j_*;

${n}_0^{C_j}$ : the number of elements in set ${N}_0^{C_j}$;
*Q_h_*: the maximum capacity of each helicopter; and
*Q_v_*: the maximum capacity of each vehicle.

(1) QBF-based EDPs selection method

Assume that *u_ij_* indicates that the MAPs *A_i_* belong to the degree of membership function of the EDP *C_j_*, $0\le{u}_{ij}\le 1$, satisfied:

(1) \begin{equation*} \sum \limits_{j=1}^m{u}_{ij}=1,\forall i=1,2,\dots, n \end{equation*}

_._

According to Bezdek [14], the objective function of the following EDPs selection can be constructed:

(2) \begin{align*} J=\sum \limits_{i=1}^n\sum \limits_{j=1}^m{\left({u}_{ij}\right)}^w{\left({d}_{ij}\right)}^2. \end{align*}

In Eq. (2), ${({d}_{ij})}^2$ represents the distance between the emergency transfer station *C_j_* and the MAP *A_i_*. This paper uses Euclidean metric, ${({d}_{ij})}^2={\Vert{A}_i-{C}_j\Vert}^2$. $\omega \in [1,\infty )$is fuzzy weighted factor. The clustering criterion of the QBF algorithm is to minimize the objective Eq. (2):

(3) \begin{equation*} \min \left\{\sum \limits_{i=1}^n\sum \limits_{j=1}^m{\left({u}_{ij}\right)}^w{\left({d}_{ij}\right)}^2\right\}. \end{equation*}

The constraint of the above objective functions is $\sum \limits_{j=1}^m{u}_{ij}=1,\forall i=1,2,\dots, n$. According to the Lagrange multiplier method, the constrained objective Eq. (3) is converted to the following unconstrained objective function:

(4) \begin{equation*} F=\sum \limits_{i=1}^n\sum \limits_{j=1}^m{\left({u}_{ij}\right)}^w{\left({d}_{ij}\right)}^2+\sum \limits_{i=1}^n{\lambda}_i\left(\sum \limits_{j=1}^m{u}_{ij}-1\right). \end{equation*}

${\lambda}_i$ represents the Lagrange multiplier of constraint (1).

The above unconstrained Eq. (4) ${u}_{ij}$, ${\lambda}_i$ and *C_j_* are biased, and the necessary conditions for minimizing as follows in Eq. (5) and Eq. (6):

(5) \begin{equation*} {u}_{ij}=\frac{1}{\sum \limits_{k=1}^m\left(\frac{d_{ij}}{d_{ki}}\right)2/\left(\omega -1\right)} \end{equation*}

(6) \begin{equation*} {C}_j=\frac{\sum \limits_{i=1}^n{\left({u}_{ij}\right)}^{\omega }{A}_i}{\sum \limits_{i=1}^n{\left({u}_{ij}\right)}^{\omega }}. \end{equation*}

For a given data set, an iterative algorithm can be used to get its specific ${u}_{ij}$ and ${C}_j$. Usually, the termination condition of the iterative algorithm is ${\max}_{ij}\big\{|{u}_{ij}^{(t)}-{u}_{ij}^{(t-1)}|\big\}<\varepsilon, 0\le \varepsilon \le 1$, where *t* represents the iteration step.

(2) QBFwCC-based EDP selection method

**Figure 1:**
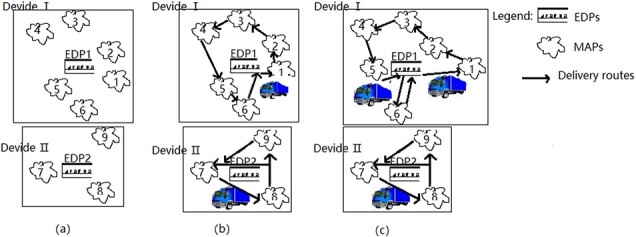
Joint transportation network of medical materials in public health emergencies.

QBF’s clustering criterion is to get the minimum value of the objective Eq. (2) that the total distance between the EDPs and the MAPs is minimized. This method focuses on the distance factor, ignoring the effective use of transportation vehicles and the effectiveness and fairness of medical supplies arriving at the MAP, resulting in unreasonable division. This unreasonable division includes two types of situations. The first type is that the capacity of the material transport vehicle is large enough to load the materials of all aid points independently in the divided area, but the last aid point in each divided area needs a longer waiting time. As shown in Figure 1b, the longer waiting time of the rescue point 6 in the division area I and the rescue point 9 in the division area II will reduce their medical utility. The other type is that the capacity of the material transport vehicle is not large enough to load the materials of all aid points independently in the divided area, which needs to increase the transportation vehicle. As shown in division area I in Figure 1c, this may result in inadequate space for transport vehicles.

In order to optimize the division of relief materials, the capacity constraints of vehicles are further considered based on QBF. In Figure 1, it is assumed that MAP 6 is assigned to EDP 2, the utility gap of medical supplies reaching the last MAP in each division and the unreasonable use of transport vehicles will be reduced. In this paper, a QBF optimization method with vehicle capacity constraints, QBFwCC, is proposed to optimize the location of EDPs and the division of MAPs.

The objective function of QBFwCC can be defined as follows:

(7) \begin{equation*} \min \left\{\sum \limits_{i=1}^n\sum \limits_{j=1}^m{\left({u}_{ij}\right)}^{\omega }{\left({d}_{ij}\right)}^2+M\sum \limits_{j=1}^m\left({Q}_v-\sum \limits_{i\in{N}^{C_j}}{d}_i Mod{Q}_v\right)\right\}. \end{equation*}

In Eq. (7), *M* represents penalty term (a large positive number) and ${Q}_v-\sum \limits_{i\in{N}^{C_j}}{d}_i Mod{Q}_v$ represents the capacity of the rest vehicles in division *j*. According to the Lagrange multiplier method, the constrained optimization problem can be converted to the following unconstrained optimization problem in Eq. (8):

(8) \begin{align*} F&=\sum \limits_{i=1}^n\sum \limits_{j=1}^m{\left({u}_{ij}\right)}^{\omega }{\left({d}_{ij}\right)}^2+M\sum \limits_{j=1}^m\left({Q}_v-\sum \limits_{i\in{N}^{C_j}}{d}_i Mod{Q}_v\right)\nonumber\\ &\quad +\sum \limits_{i=1}^n{\lambda}_i\left(\sum \limits_{j=1}^m{u}_{ij}-1\right). \end{align*}

### 2.3. Optimization model of medical material delivery routes based on clustering

The QBF or QBFwCC method could be used to obtain the location of the emergency transit point and the medical rescue point covered by each emergency transit point. In this paper, a medical material delivery route optimization model based on clustering is established to determine the specific delivery route from the emergency transit point to each medical rescue point.

The new symbol definitions are proposed below:

${K}^{C_j}$ : the medical vehicles collection of emergency transit point ${C}_j$;

${d}_{L{C}_j}$ : the distance from LCDH to emergency transit point *Cj*, *j* = 1,2,…*m*; and

${d}_{il}^{C_j}\forall j\in \{1,2,\dots, m\}\forall i\in \{1,2,\dots, m\}i,l\in{N}_0^{C_j}j,l\in{N}_0^{C_j}$ : the distance between the emergency transit point ${C}_j$ and the covered medical rescue points or the distance between the rescue points;

${v}_h$ : the transport speed of the helicopter;

${v}_v$ : the transport speed of the vehicle; and

${q}_{ilk}^{C_j}$ : the residual medical supplies of delivery vehicle $k$ from rescue point ${A}_i$ to rescue point ${A}_l$.

The decision variables of medical supplies delivery routes optimization model based on clustering are as follows:

${x}_{ilk}^{C_j}$ is a binary variable, ${x}_{ilk}^{C_j}$ = 1 represents vehicle $k$ from rescue point ${A}_i$ to rescue point ${A}_l$, $\forall j\in \{1,2,\dots, m\}$, $i,l\in{N}_0^{C_j}$, $k\in{K}^{C_j}$,otherwise ${x}_{ilk}^{C_j}$=0;

${u}_{ik}^{C_j}$ is a binary variable, ${u}_{ik}^{C_j}$ = 1 represents that rescue point ${A}_i$ is served by vehicle $k$, $\forall j\in \{1,2,\dots, m\}$, $i\in{N}_0^{C_j}$, $k\in{K}^{C_j}$, otherwise ${u}_{ik}^{C_j}$=0.

In order to simplify without loss of generality, the flight time of helicopter from the distribution center to the transit point and the transport time from the transit point to the rescue point are mainly considered in this paper, the objective of the dual mode combined transport is set as follows:

(9) \begin{align*} \min \sum \limits_{j=1}^m\left(\frac{d_{L{C}_j}}{v_h}+\sum \limits_{k=1}^{k{C}_j}\sum \limits_{i=1}^{n_0^{C_j}}\sum \limits_{l=1,l\ne i}^{n_0^{C_j}}\frac{d_{il}^{{\mathrm{C}}_j}}{v_v}{x}_{il k}^{C_j}\right). \end{align*}

$\frac{d_{L{C}_j}}{v_h}$ represents the flight time of the helicopter from LCDH to emergency transit point ${C}_j$ and $\sum \limits_{k=1}^{k{C}_j}\sum \limits_{i=1}^{n_0^{C_j}}\sum \limits_{l=1,l\ne i}^{n_0^{C_j}}\frac{d_{il}^{{\mathrm{C}}_j}}{v_v}{x}_{il k}^{C_j}$ represents the total transport time of the vehicle from transit point *Cj* to its covered rescue point. Considering the constraint conditions of this goal, optimization model of medical material delivery routes based on clustering is constructed as follows:

(10) \begin{equation*} s=\sum \limits_{i=1}^n{d}_i=\sum \limits_{j=1}^m\sum \limits_{k=1}^{k{C}_j}\sum \limits_{i=1}^{n_0^{C_j}}\sum \limits_{l=1,l\ne i}^{n_0^{C_j}}{d}_i{x}_{ilk}^{C_j} \end{equation*}

(11) \begin{equation*} \sum \limits_{k=1}^{k{C}_j}\sum \limits_{i=1}^{n_0^{C_j}}\sum \limits_{l=1,l\ne i}^{n_0^{C_j}}{d}_i{x}_{ilk}^{C_j}\le{Q}_h \end{equation*}

(12) \begin{equation*} \sum \limits_{k=1}^{k^{C_j}}{u}_{ik}^{C_j}=1 \end{equation*}

(13) \begin{equation*} \sum \limits_{k=1}^{k^{C_j}}{u}_{0k}=\sum \limits_{k=1}^{k^{C_j}}{u}_{k0}\le{k}^{C_j} \end{equation*}

(14) \begin{equation*} \sum \limits_{h=1,i\ne h}^{n_0^{C_j}}{x}_{hik}^{C_j}=\sum \limits_{l=1,i\ne l}^{n_0^{C_j}}{x}_{ilk}^{C_j}={u}_{ik}^{C_j} \end{equation*}

(15) \begin{equation*} \sum \limits_{i=1}^{n^{C_j}}{d}_i\times{u}_{ik}^{C_j}-\sum \limits_{l=1}^{n^{C_j}}{q}_{0 lk}^{C_j}=0 \end{equation*}

(16) \begin{equation*} {q}_{ilk}^{C_j}\le{Q}_v\times{x}_{ilk}^{C_j} \end{equation*}

(17) \begin{align*} {x}_{ilk}^{C_j}&=\left\{0,1\right\},{u}_{ik}^{C_j}=\left\{0,1\right\},i\in \left\{1,2,\dots, m\right\},\nonumber\\ m&\le H\ h,j,l\in{N}_0^{C_j},k\in{K}^{C_j} \end{align*}

Eq. (10) represents that the amount of medical supplies allocated to all MAP is equal to the available quantity of LCDH current medical supplies; Eq. (11) represents that the total volume of medical supplies delivered to emergency trait point *C_j_* does not exceed the maximum capacity of helicopter; Eq. (12) represents that each medical rescue point is only served once by a transport vehicle; Eq. (13) represents that each transport vehicle starts from its corresponding emergency transit point and eventually returns to the emergency transit point; Eq. (14) represents that the vehicles arriving at the MAP *A_i_* should leave from this MAP; Eqs. (15) and (16) represent that each delivery vehicle cannot carry medical supplies exceeding its maximum capacity; and Eq. (17) defines the value range of symbols and variables.

### 2.4. Performance measurement indicators of multimodal transport

The transport of emergency supplies not only pursues the transport efficiency, but also pays attention to the effective use of vehicles, the effectiveness and fairness of arriving supplies, etc. Because of the complexity of solving multi-objective problem, the duration of medical supplies delivery was taken as the optimization goal, and the following three performance measures were constructed in the paper:

(1) Total extra capacity (TEC) of vehicle,

(18) \begin{equation*} TEC=\sum \limits_{j=1}^m\left({Q}_v-\sum \limits_{i\in{N}^{C_j}}{d}_i Mod{Q}_v\right). \end{equation*}

The smaller the total residual capacity of the vehicle, the higher the efficiency of the vehicle.

(2) Average waiting time (AWT),

(19) \begin{equation*} AWT=\frac{\sum \limits_{j=1}^m\left(\frac{d_{L{C}_j}}{v_h}+\sum \limits_{k=1}^{k^{C_j}}\sum \limits_{i=1}^{n^{C_j}}\sum \limits_{l=1,l\ne i}^{n^{C_j}} ari{v}_i^{C_j}\right)}{n}. \end{equation*}

$ariv\frac{C_j}{i}$ represents the time when the medical supplies arrive at medical rescue point *Ai*. The shorter the average waiting time, the more effective the medical supplies.

(3) Best waiting time (BWT),

(20) \begin{equation*} BWT=\underset{j=1}{\overset{m}{\max }}\left(\frac{d_{L{C}_j}}{v_h}+\underset{k\in{K}^{C_j}}{\max}\sum \limits_{i=1}^{n_0^{C_j}}\sum \limits_{l=1,l\ne i}^{n_0^{C_j}}{\theta}_{il}^{C_j}{t}_{il}^{C_j}{x}_{il k}^{C_j}\right). \end{equation*}

To some extent, the best waiting time reflects the difference of material arriving time. The shorter the indicator, the fairer the delivery.

## 3. SOLUTION

### 3.1. Heuristic algorithm based on QBF emergency transition point selection and division

The input of the QBF algorithm:

*n*: the number of MAPs;

*A_j_* (*j* = 1,2,…,*n*): the location of MAPs;

*m*: the number of emergency transfer points should be selected;

*w*: the fuzzy weighting coefficient of QBF; and

$\varepsilon$ : the iteration termination threshold of QBF.

The output of the QBF algorithm:

*C_i_*(*t*): the location of the selected emergency transit point;

*U*  ^(*t*)^: the final membership matrix;

*J*  ^(*t*)^: the objective function value of QBF; and

${N}^{C_i}$ : the collection of MAPs covered by emergency transit point *C_i_*.

The specific algorithm steps are as follows:

Step 1: Initialization parameters *n*, *A_j_*, *m*, *w* and $\varepsilon$, the number of bacteria, migration times, reproduction times, swimming times and migration probability are mapped in the algorithm.

Step 2: Randomly generate an initial membership matrix,${U}^{(0)}={[{u}_{ij}]}_{m\times n}$, where $0\le{u}_{ij}\le 1$, $\sum \limits_{i=1}^m{u}_{ij}=1,\forall j=1,2,\dots, n;$.

Step 3: Use Eq. (6) to calculate the location of the emergency transit point *C_i_*(*t*), where *t* represents the iteration step, *i* = 1,2,…,*m*.

Step 4: Use ${J}^{(t)}=\sum \limits_{j=1}^n\sum \limits_{i=1}^m{({u}_{ij}(t))}^{\omega }{({d}_{ij})}^2$ and ${({d}_{ij})}^2={\big\Vert{A}_j-{C}_i(t)\big\Vert}^2$ to calculate the objective function value. If ${\max}_{ij}\big\{|{u}_{ij}^{(t)}-{u}_{ij}^{(t-1)}|\big\}<\varepsilon$, terminate the iteration, record the values of *C_i_*(*t*), *U*^(*t*)^ and *J*^(*t*)^ and go to step 6.

Step 5: Use Eq. (5) to calculate the new membership matrix *U*^(*t* + 1)^，let *t* = *t* + 1, and turn to step 2.

Step 6: According to the final membership matrix *U*^(*t*)^, the maximum membership of each medical assistance point *A_j_* belonging to the emergency transit point *C_i_*(*t*) was calculated and the set of medical assistance points covered by each emergency transit point *C_i_*(*t*) was determined, namely${N}^{C_i}$, output *C_i_*(*t*), *U*^(*t*)^, *J*^(*t*)^ and${N}^{C_i}$*.*

### 3.2. Heuristic algorithm based on QBFwCC emergency transition point selection and division

This section proposes the method of emergency transit point selection and medical assistance point division based on QBFwCC.

Input to the QBFwCC algorithm:

*n*: the number of MAPs;

*A_j_* (*j* = 1,2,…,*n*): the location of MAPs;

*m*: the number of emergency transfer points to be selected;

*w*: the fuzzy weighting coefficient of QBF;

$\varepsilon$ : the iteration termination threshold of QBF;

*Q_v_*: maximum capacity of transport vehicle; and

*d_j_*: the amount of medical supplies distributed by the MAP *A_j_*.

The output of the QBFwCC algorithm:

*C_i_*(*t*): the location of the selected emergency transfer point; and

${N}^{C_i}$ : the adjusted division of medical assistance points.

The specific steps are as follows:

Step 1: Initialization parameters *n*, *A_j_*, *m*, *w*,$\varepsilon$,*Q_v_* and *d_j_*, the number of individual bacteria, migration times, reproduction times, swimming times, migration probability, chemotaxis times and randomly generated vector of bacterial individuals are mapped in the algorithm;

Steps 2–5: Same steps as QBF algorithm;

Step 6: According to the membership matrix *U*^(*t*)^, the maximum membership of each MAP *A_j_* belonging to the emergency transit point *C_i_*(*t*) was calculated, the set of medical assistance points covered by each emergency transit point *C_i_*(*t*) was determined, namely${N}^{C_i}$, and the values of *C_i_*(*t*), *U*^(*t*)^, *J*^(*t*)^ and${N}^{C_i}$were recorded, at the same time a state variable set flag = {*C_i_*,*i* = 1,2,…,*m*} was set, go to step 7.

Step 7: ${C}_{extra}^i={Q}_v-\sum \limits_{j\in{N}^{C_i}}{d}_j Mod{Q}_v$ is used to calculate the remaining capacity of the vehicle in each division. If the number of elements in the state variable set flag is equal to 1, record the adjusted${N}^{C_i}$, go to step 10.

Step 8: The division with the largest remaining capacity ${C}_{extra}^i$ in the state variable and flag is determined, according to the size of the 2nd membership degree of the medical assistance points in the division, several rescue points are sequentially removed from them, so that the total amount of medical supplies allocated to the remaining medical assistance points in the division divided by the vehicle capacity *Q_v_* is exactly the largest; remove the division from the state variable set flag.

Step 9: Divide the medical assistance points selected in step 8 to the division with the second highest of membership and go to step 7.

Step 10: Output *C_i_*(*t*) and adjusted ${N}^{C_i}$.

## 4. COMPUTATIONAL EXPERIMENTS

### 4.1. Experiment data

To verify the effectiveness of the method proposed in this paper, experimental data are set as follows in this section. When COVID-19 broke out in an area, the emergency management department set up 50 medical rescue points in the area, and the horizontal and vertical coordinates of each point were randomly generated within [0,300] (unit: km). The specific data are shown in Table 1.The materials needed for medical assistance should be urgently gathered from the field to the LCDH in the region with the coordinates set at (150,150) and they should be sent to each medical assistance point as soon as possible. The amount of materials allocated for each MAP is randomly generated in the interval [300,600] (unit: pieces).

**Table 1:** *Coordinates of MAPs and distribution of supplies*.

Hub	Abscissa	Ordinate	Allocation amount	Hub	Abscissa	Ordinate	Allocation amount
1	194	174	510	26	87	285	316
2	5	129	365	27	149	212	534
3	281	273	306	28	27	51	319
4	156	69	526	29	228	195	446
5	164	204	274	30	59	62	473
6	209	198	521	31	281	197	522
7	96	41	504	32	71	288	352
8	123	161	484	33	291	23	499
9	128	41	317	34	165	59	529
10	83	110	416	35	255	218	259
11	48	234	461	36	170	179	575
12	93	42	515	37	275	18	353
13	59	210	371	38	99	135	506
14	78	71	588	39	186	51	529
15	132	111	468	40	290	269	446
16	195	162	593	41	54	111	298
17	167	210	354	42	234	284	276
18	290	125	284	43	14	75	466
19	233	44	558	44	17	170	521
20	12	195	420	45	147	72	600
21	80	200	299	46	185	173	448
22	156	230	326	47	2	95	314
23	194	174	510	48	87	285	316
24	5	129	365	49	149	212	534
25	281	273	306	50	27	51	319

### 4.2. Results based on QBF

#### 4.2.1 Selection and division of emergency transit points based on QBF

**Figure 2:**
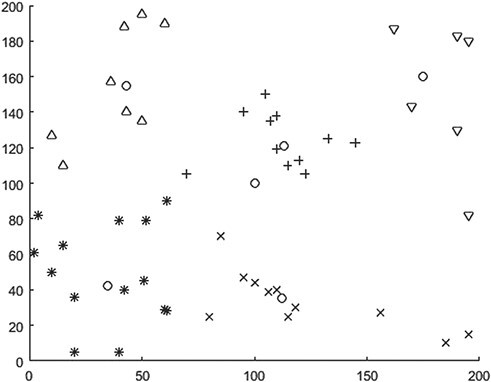
Selection and division of emergency transit points based on QBF.

In this paper, the QBF algorithm in Section 3.1 is implemented based on MATLAB R2019b. The relevant parameters of the algorithm are set as follows: QBF fuzzy weighted coefficient w = 2, iterative algorithm termination threshold $\varepsilon$=1*10–5 and the maximum number of iterations is set as 300. After 39 iterations, the algorithm reached the termination condition and the final QBF objective function value was 34878.31. The results of emergency transfer point selection and medical rescue point division were shown in Figure 2 (the box represented LCDH, the five circles represented the selected emergency transfer point and the other five different shapes represented the division of medical rescue point).

Table 2 lists the specific locations of the selected emergency transfer points and the cluster of rescue points corresponding to each transfer point. Combined with the material distribution of each divided MAP in Table 1, the residual vehicle capacity of each divided transit point (assumed vehicle capacity Qv = 2000 here) can be obtained. The residual capacity is expressed as ${C}_{extra}^i$ in Table 2. As can be seen from Table 2, there is a large amount of surplus capacity in subdivisions 1–4. Although the QBF method minimizes the total distance between the emergency transfer point and the MAP, it only considers the distance criterion and lacks constraints on the vehicle capacity of each division, which tends to lead to the imbalance of the remaining vehicle capacity at the transfer point.

#### 4.2.2 Distribution route results based on QBF

**Table 2:** *Selection and division of emergency transit points based on QBF*.

EDPs	Abscissa	Ordinate	${N}^{C_i}$	${n}^{C_i}$	$\sum \limits_{j\in{N}^{C_i}}{d}_j$	${C}_{extra}^i$
EDP 1	117.9331	37.9127	{1,7,12,18,22,27,36,37,40,42,48}	11	44.79	15.21
EDP 2	117.3180	123.9910	{4,8,9,11,19,20,25,30,32,39,49}	11	42.20	17.80
EDP 3	30.6857	47.7180	{2,5,10,13,15,17,26,28,31,33,41,44,46,50}	14	49.87	10.13
EDP 4	38.3527	153.4735	{3,14,16,23,24,29,35,47}	8	24.10	14.90
EDP 5	176.9927	158.0736	{6,21,34,38,43,45}	6	17.45	2.55

**Figure 3:**
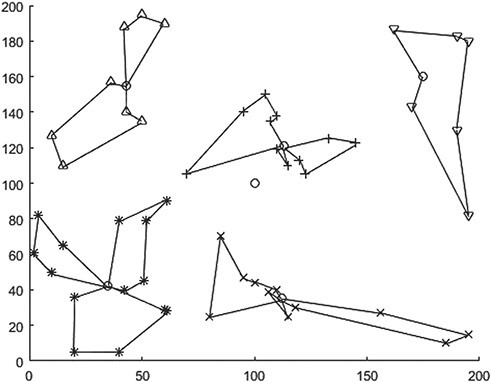
Distribution routes selected by emergency transit points based on QBF.

After the location of the emergency transit point is selected, the follow-up problem is to plan the transit route between the transit point and the MAP and to design the optimization model of the transit route. The vehicle capacity of Qv is set as 20 m3, helicoper flying speed of Vh is 500 km/h, the vehicle running speed of Vv is 100 km/h. The calculation results are shown in Figure 3 and Table 3. The total transport time is 1509.07 min, the number of medical assistance vehicles is 12 (the number of helicopters is equal to the number of emergency transit points, set as 5), the total vehicle remaining capacity is 60.59 m3, the average waiting time is 85.92 min and the maximum waiting time is 272.56 min.

It can be seen that the QBF method and algorithm can obtain the location of the emergency transit point and the corresponding material delivery route. However, due to the lack of constraints on the remaining vehicle capacity in the division, the division may be unreasonable. For example, in the red transport route in Figure 3, there is a large idle vehicle capacity, and the remaining capacity is 11.31 and 11.48, respectively. Similar residual capacity also exists in other routes.

### 4.3. Results based on QBFwCC

#### 4.3.1 Selection and division of emergency transit points based on QBFwCC

**Figure 4:**
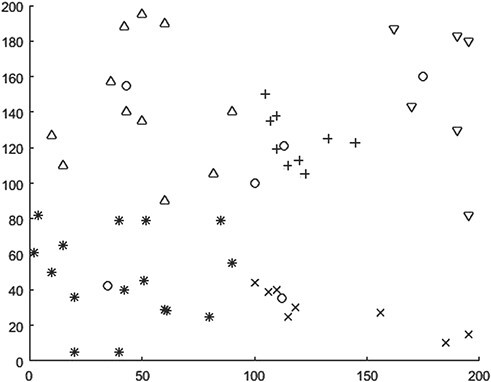
Selection and division of emergency transit points based on QBFwCC.

Considering the limitations of the QBF method, this section runs Matlab R2019b to implement the QBFwCC algorithm in Section 3.2 on the basis of it. The final QBFwCC adjustment results are shown in Figure 4 and Table 4. By comparing Tables 4 and 2, it can be found that the adjustment of MAP division by QBFwCC reduces the capacity of the remaining vehicles in each division. But the QBFwCC still has not overcome the uneven number of MAPs in each division.

#### 4.3.2 Delivery route based on QBFwCC

**Figure 5:**
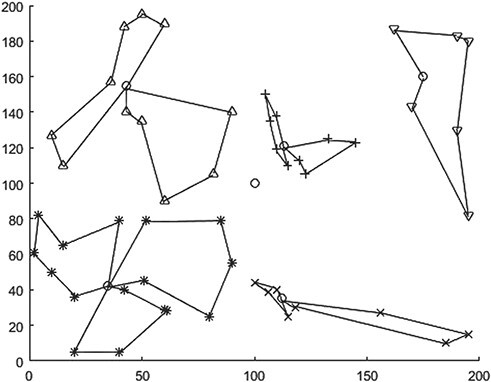
Distribution routes selected based on QBFwCC emergency transit points.

Set the same parameters as in Section 4.1: vehicle capacity as Qv = 20 cubic meters, helicopter flying speed as VH = 500 km/h and vehicle running speed as VV = 100 km/h. The calculation results are shown in Figure 5 and Table 5. The total transport time is 1577.95 min, the number of medical assistance vehicles is 10 and the total vehicle residual capacity is 20.72 cubic meters, which is nearly 40 cubic meters less than that based on QBF method (the capacity of 2 vehicles). The average waiting time is 90.05 min, and the maximum waiting time is 272.46 min.

**Table 3:** *Delivery routes selected based on QBF emergency transit points*.

EDPs	Delivery routes	Vehicle surplus capacity (m^3^)	Total waiting time for supplies		Longest waiting time for supplies	
			LCDH->EDP	EDP->MAP	LCDH->EDP	EDP->MAP
	0->37->1->0	11.31	12.93	28.75	12.93	28.72
EDP 1	0->22->36->40->42->0	3.84	12.93	362.57	12.93	161.15
	0->12->18->48->7->27->0	0.06	12.93	470.11	12.93	135.36
	0->4->19->32->9->0	2.75	5.92	156.15	5.92	89.58
EDP 2	0->11->30->25->20->8->0	3.57	5.92	420.98	5.92	124.04
	0->39->49->0	11.48	5.92	23.97	5.92	28.01
	0->15->10->26->2->31->0	2.32	17.36	369.91	17.36	142.61
EDP 3	0->33->17->13->41->44->0	0.99	17.36	263.74	17.36	132.24
	0->46->50->5->28->0	6.82	17.36	203.05	17.36	107.46
	0->29->35->3->0	12.16	16.32	159.8	16.32	99.35
EDP 4	0->14->23->47->24->16->0	2.74	16.32	336.19	16.32	135.14
EDP 5	0->38->21->34->43->6->45->0	2.55	19.29	803.42	19.29	253.57

**Table 4:** *Selection and division of emergency transit points based on QBF*.

EDPs	Abscissa	Ordinate	${N}^{C_i}$	${n}^{C_i}$	$\sum \limits_{j\in{N}^{C_i}}{d}_j$	${C}_{extra}^i$
EDP 1	117.9331	37.9127	{1,7,12,18,22,27,36,37,40,42,48}	11	44.79	15.21
EDP 2	117.3180	123.9910	{4,8,9,11,19,20,25,30,32,39,49}	11	42.20	17.80
EDP 3	30.6857	47.7180	{2,5,10,13,15,17,26,28,31,33,41,44,46,50}	14	49.87	10.13
EDP 4	38.3527	153.4735	{3,14,16,23,24,29,35,47}	8	24.10	14.90
EDP 5	176.9927	158.0736	{6,21,34,38,43,45}	6	17.45	2.55

**Table 5:** *Delivery routes selected based on QBF emergency transit points*.

EDPs	Delivery routes	Vehicle residual capacity	Total waiting time for supplies		Longest waiting time for supplies	
			LCDH->EDP	EDP->MAP	LCDH->EDP	EDP->MAP
	0->37->1->0	11.31	12.93	28.75	12.93	28.72
EDP 1	0->22->36->40->42->0	3.84	12.93	362.57	12.93	161.15
	0->12->18->48->7->27->0	0.06	12.93	470.11	12.93	135.36
	0->4->19->32->9->0	2.75	5.92	156.15	5.92	89.58
EDP 2	0->11->30->25->20->8->0	3.57	5.92	420.98	5.92	124.04
	0->39->49->0	11.48	5.92	23.97	5.92	28.01
	0->15->10->26->2->31->0	2.32	17.36	369.91	17.36	142.61
EDP 3	0->33->17->13->41->44->0	0.99	17.36	263.74	17.36	132.24
	0->46->50->5->28->0	6.82	17.36	203.05	17.36	107.46
	0->29->35->3->0	12.16	16.32	159.8	16.32	99.35
EDP 4	0->14->23->47->24->16->0	2.74	16.32	336.19	16.32	135.14
EDP 5	0->38->21->34->43->6->45->0	2.55	19.29	803.42	19.29	253.57

## 5. Conclusion

In the case of major public health events, the distance between the supply node and the demand node is too far, and it is difficult for the key roads to transport materials to the epidemic area by vehicles in time. A two-stage combined transportation method of medical supplies based on clustering is proposed in this paper. The experimental results verify the effectiveness of the proposed method and algorithm and draw the following conclusions: (1) Although the results of conventional QBF division can minimize the total distance between the emergency transit point and the medical assistance point, due to the lack of constraints on the vehicle capacity in each division, a larger remaining capacity may be generated. (2) Through the adjustment of QBFwCC to the division of medical assistance points, the remaining vehicle capacity in each division is reduced, which can effectively reduce the number of vehicles used when the total delivery time and average waiting time are slightly delayed.

Although the model and algorithm proposed in this article can effectively select emergency transit points and provide certain decision support for the actual joint transportation of emergency materials, further research directions should be focused, such as how to determine the optimal number of emergency transit points according to the disaster scenario and how to overcome the imbalance in the number of medical assistance points in the division. At the same time, the model constructed in this article mainly has the following applicable conditions: the emergency resources of joint transportation are limited to small-sized critical emergency materials, and are not suitable for the transportation of large-volume materials, such as large-scale medical equipment; and the loading and unloading capacity of the distribution center and transfer point is required to be relatively sufficient.

## Declaration of competing interest

None. This research did not receive any specific grant from funding agencies in the public, commercial or not-for-profit sectors.
